# Aging in the Aftermath of Adversity: Later-Life Impact of Institutional Child Abuse and Disclosure

**DOI:** 10.1093/geroni/igab046.283

**Published:** 2021-12-17

**Authors:** Shauna Rohner, Andreas Maercker, Alan Carr, Myriam Thoma

**Affiliations:** 1 University of Zurich, Zürich, Zurich, Switzerland; 2 University of Zurich, University of Zürich, Zurich, Switzerland; 3 University College Dublin, Dublin, Dublin, Ireland; 4 University of Zurich, Zurich, Zurich, Switzerland

## Abstract

Until the 1990’s in Ireland, many children in institutional care experienced abuse and neglect, with lasting negative effects, including trauma symptoms and psychopathology. While trauma disclosure can be important for recovery, findings are inconsistent and often lack consideration of wider social and interpersonal contexts. As survivors of this historical adversity enter later-life stages, research is needed on the long-term impact and to clarify the role of disclosure. Therefore, this study aimed to examine the later-life impact of institutional child abuse on health and well-being, and the role of trauma disclosure and socio-interpersonal contexts in an older adult sample. Qualitative semi-structured interviews (60-120 minutes) were conducted with 17 Irish older adults, aged 50-77 years (mean age=60.7 years), who experienced childhood institutional abuse. Audio-recorded interviews were transcribed and analysed using Framework Analysis. Themes for ‘childhood and related later-life adversity’ included detrimental perceptions and interactions, re-exposure and reminders, failure of system and society, and cycle of abuse. Disclosure themes included successful, unsuccessful, and non-disclosure, as well as evidence of socio-interpersonal interactions (e.g., non-disclosure influenced by shame or fear, compounded by socio-cultural values, (lack of) social acknowledgment, or the power of the church in society). Results suggest that childhood institutional abuse can have long-term negative impacts into later life, including social, psychological, physical health, and socio-economic aspects. Disclosure results emphasize the need to consider the complex social, cultural, and interpersonal contexts within which an individual is embedded. This may enhance understanding and facilitate targeted health and social care services for this older adult population.

